# The Effect of Tannins on Mediterranean Ruminant Ingestive Behavior: The Role of the Oral Cavity

**DOI:** 10.3390/molecules16042766

**Published:** 2011-03-25

**Authors:** Elsa Lamy, Harshadrai Rawel, Florian J. Schweigert, Fernando Capela e Silva, Ana Ferreira, Ana Rodrigues Costa, Célia Antunes, André Martinho Almeida, Ana Varela Coelho, Elvira Sales-Baptista

**Affiliations:** 1 Instituto de Ciências Agrárias e Ambientais Mediterrânicas (ICAAM), Universidade de Évora, Évora, Portugal; 2 Escola Superior de Hotelaria e Turismo do Estoril (ESHTE), Estoril, Portugal; 3 Institute of Nutritional Science, University of Potsdam, Nuthetal (OT Bergholz-Rehbrücke), Germany; E-Mails: rawel@uni-potsdam.de (H.R.); fjschwei@uni-potsdam.de (F.J.S.); 4 Departamento de Biologia, Universidade de Évora, Évora, Portugal; E-Mail: fcs@uevora.pt; 5 Departamento de Química, Universidade de Évora, Évora, Portugal; E-Mail: acrc@uevora.pt; 6 Centro de Neurociências e Biologia Celular (CNBC), Universidade de Coimbra, Coimbra, Portugal; E-Mail: cmma@uevora.pt; 7 Instituto de Investigação Científica Tropical (IICT) & CIISA – Centro Interdisciplinar de Investigação em Sanidade Animal. CVZ - Centro de Veterinária e Zootecnia, Faculdade de Medicina Veterinária, Lisboa, Portugal; E-Mail: aalmeida@fmv.utl.pt; 8 Instituto de Tecnologia Química e Biológica/Universidade Nova de Lisboa (ITQB/UNL), Oeiras, Portugal; E-Mail: varela@itqb.unl.pt; 9 Departamento de Zootecnia, Universidade de Évora, Évora, Portugal; E-Mail: elsaba@uevora.pt

**Keywords:** polyphenols, diet selection, ruminants, salivary proteins, tannin-protein interaction

## Abstract

Sheep, cattle and goat are domestic ruminants of significant economic interest in the Mediterranean region. Although sharing the same pasture ranges, they ingest different plants and plant parts and, consequently different levels of tannins. This suggests an ability to detect and adapt ingestion according to animal physiological limits of tolerance for plant secondary metabolites. This review will detail the effects of dietary tannins on feeding behavior, and the role of the oral cavity in this process, with focus on such ruminant species. The role of salivary protein profile in tannin perception in the oral cavity, and as a defense mechanism, will be discussed.

## 1. Introduction

In animal production, nutrition is one of the most important factors, being determinant to productive performance. Consequently, the understanding of ingestive behavior, and particularly dietary choices and adaptation to pastures, is of extremely importance in livestock management. Differences among free-grazing ruminant species, concerning food selection, allow an efficient pasture use at the habitat scale. Additionally, an effective and sustainable animal management, as well as ecological and environmental aspects, would benefit from a well founded knowledge on animal-plant interactions.

Since the 1950s the ingestive behavior of ruminants fed both indoors and on pastures has been extensively studied. Intake is influenced primarily by hunger, which is distressing, and by satiety, which is generally pleasant [1]. However, food intake is not so simple. In fact, it is a complex behavior, which involves simultaneously homeostatic and hedonic aspects. On one hand, factors related to the biological requirements for energy and nutrients modulate the beginning and the end of an ingestion episode, also influencing the type of feed chosen. On the other hand, affective factors, linked to past experiences, will affect the likeness and the consumption of a particular food item. This complexity is further increased by the interaction of these homeostatic and hedonic aspects [2,3]. 

Among the substances present in herbivores’ diets, plant secondary metabolites (PSMs) have an ecological function of plant protection from the attack of pathogens and consumption by herbivores. PSMs comprise a wide range of chemicals, such as terpenes, alkaloids, oxalates, saponins and tannins. In ruminant nutrition, it has been shown that the levels of tannins present in food items represent a major component of food choice [4]. 

The inverse relation between high tannin levels in forage and palatability, voluntary intake, digestibility and nitrogen retention has long been established in several herbivores [5,6,7]. Reduced palatability, low evacuation rate of the digested material out of the rumen and toxicity are factors that were considered as an explanation for the negative effects of tannins on ruminants feed intake [8,9]. However, several ruminant species seem to tolerate (or even prefer) considerable amounts of tannins in their diets [10,11].

The discrepancy in the tolerance to tannins among herbivores, in general, and ruminants, in particular, can be related to different defense mechanisms that each species present to PSMs. The oral cavity plays an important role in the process of tannin ingestion, both by being the place of detection of these plant compounds, and through the presence of salivary proteins which act as defense mechanisms. In this review, we present a summary of the current knowledge on the influence of dietary tannins in domestic ruminant species usually exploited in Mediterranean region. We will discuss in detail the importance of tannin-salivary protein interaction and the role of salivary protein profile in animal ingestive behavior.

## 2. Food Selection and Grazing Ecology

Understanding ruminant foraging behavior requires a perspective in which herbivores are seen as intermediate players in trophic chains that flexibly adapt their behavior to balance gains and losses imposed by resource limitation [12]. Plants are generally a diffuse source of nutrients, and large amounts of material need to be ingested to maintain the metabolism of herbivorous [13]. To meet their requirements for maintenance, growth and reproduction, ruminants are faced with an optimization problem and need to make a complex set of decisions [14], including when and where to graze and how much herbage to consume, in order to maximize nutrient intake while minimizing potentially toxic metabolites. The heterogeneity of the environment in which they are foraging, both at the plant community and chemical levels, provide diversity that contribute to adaptive foraging behavior. Therefore, habitat selection can vary according to the ability of animals to eat, digest and detoxify foods [15].

Hofmann [16] used this eco-physiological adaptation concept to explain the diversity in ruminant dietary patterns, classifying them in three overlapping morpho-physiological feeding types: grazers, browsers (concentrate selectors), and intermediate (opportunistic) feeders. Although this classification is widely accepted, it has also been criticized due to several flaws, including the lack of evidence for some of Hofmann’s assumptions (reviewed in [17]). Nevertheless it has been used to conceptualize adaptive strategies in forage selection, avoidance and utilization. In general, grazers have a diet based on monocotyledons, which are fibrous, having high amounts of cellulose and lignin. Browsers select for fresh, juicy foliage, forbs and other dicotyledonous matter with a high proportion of easily digestible plant material relatively rich in energy and protein, especially plant cell contents and little plant cell wall constituents (fiber). The drawback of browsers’ diets is their high levels of PSMs, namely tannins. Intermediate feeders can behave as both browsers and grazers seasonally, depending on the vegetable species available.

Despite the general idea that tannins are only found in plant species from tropical or arid/semi-arid areas, they are also found in other regions. The Mediterranean vegetation is dominated by evergreen shrubs and sclerophyllous trees adapted to the distinctive climatic regime of summer drought and cool moist winters [18]. Most evergreen oak woodlands (known as *montado* in Portugal and *dehesa* in Spain), and shrublands (e.g Cystaceae, Ericaceae) are characterized by high levels of PSMs. Cattle, sheep, and goats are domestic ungulates of significant relevance to Mediterranean animal production, largely based on extensive systems with free-grazing animals using native vegetation. As they choose different plant species and plant parts, common grazing in the same pasture areas is possible, maximizing its use. Although cattle and sheep are seen as grazers, whereas goats behave as intermediate feeders [16], these classifications are not consensual and some authors consider that both sheep and goats should be considered intermediate feeders [19]. These variations in feeding behavior, even for the same species, is not surprising and could be accounted to the high level of genetic diversity among the different breeds of goats [20] and sheep [21] and the vast range of ecosystems they are bred. Whatever the classification, there is a general consensus in considering that goats eat proportionally more browsing material than sheep. Goats usually have the capacity of adapting their ingestive behavior to food items available, and select diet compounds in order to maintain the proportions between nutrients and PSMs relatively constant throughout the year [22]. Although goats prefer vegetable species that present high nutritive values, when available, they may perform well in environments that are inadequate to most ruminants [23]. Under natural conditions, goats are generally active, selective, walk long distances in search for feed and choose a diet based in foraging grass. However, under resource limiting conditions goats will become heavy browsers of trees and shrubs and less discriminating in their grazing habits, due to the reduced supply of available herbage. On the other hand, sheep are less selective and use pasture more effectively when quality plants are available, but in harsh environments their productivity decreases greatly [24]. On Mediterranean woodland and brushland, browse constitutes more than 40% of goats’ diets [22,25], whereas for sheep the amount is considerable lower [25,26]. Goats that are allowed to graze freely in such environments will organize their feeding behavior to select dietary components in such a way that the available protein, neutral-detergent fiber (NDF) and condensed tannins in the total diet remains relatively constant throughout the year [22].

Most of the referred differences on diet selection, between sheep and goats, can be attributed to genetic differences that account for differences in innate sensory ability, and other morpho-physiological characteristics. Also past experiences responsible for learning mechanisms, through foraging consequences including post-ingestive feedback, trial-and-error and social learning and spatial memory, among others appear to be involved in food preferences (for a more comprehensive review please refer to [27]). However, the complexity resulting from combining biological predisposition with experiential input are always important determinants of diet selection. The knowledge of the phenomena at the level of the oral cavity can contribute to the comprehension of both strands.

## 3. Tannins and Its Relation to the Choice of Food

### 3.1. Tannin structure and the nature of their interactions with proteins

The word tannin refers to a heterogeneous group of polymeric phenolic compounds usually present in plants. This word was originally used to describe plant extracts used to tan animal leather [28]. The use of tannin-rich foods in animal production is a matter of interest, since through the years it has been demonstrated that tannins may exert both favorable and detrimental effects, when consumed by ruminants [29]. The ability of these PSMs to form strong complexes with proteins is one of the main aspects of their anti-nutritive effects. These complexes are more or less stronger depending both on the tannin and proteins properties [28,30].

**Figure 1:**
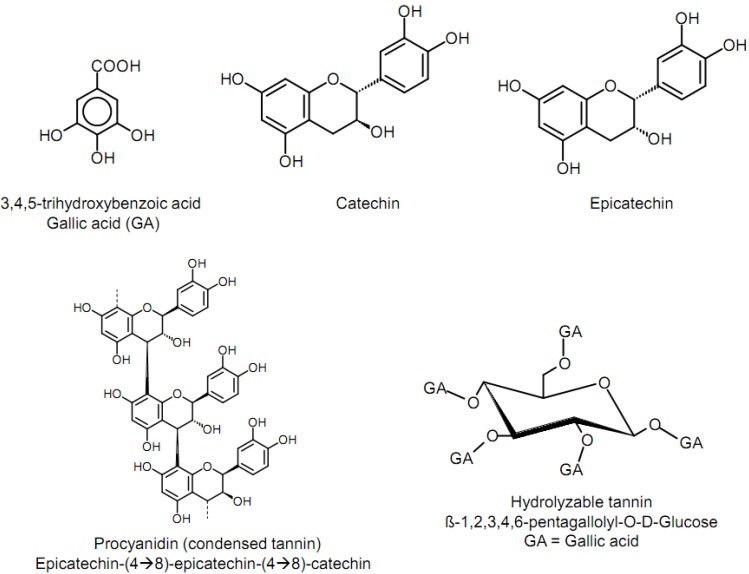
Typical Structures of hydrolyzable and condensed tannins.

Structurally, tannins possess 12–16 phenolic groups and 5–7 aromatic rings per 1,000 units of relative molecular mass. Hydrolysable tannins (Figure 1) represent esters of phenolic acids (generally gallic acid as in gallotannins or other phenolic acids derived from the oxidation of galloyl residues as in ellagitannins) and a polyol, usually glucose [31]. The galloyl groups can be further esterified or oxidatively crosslinked to yield more complex hydrolysable tannins. The molecular masses range from 300–5,000 Da. Many studies have proved that small flavonoids are more beneficial to health when compared to large ones [32,33] and the research goals to obtain highly active biological small molecules from large tannins have been conducted.

The most important studied group of polyphenols is that of the condensed tannins also termed proanthocyanidins (PAs, Figure 1), which are among the most abundant polyphenols in the plant kingdom. A typical property of PAs is that they yield anthocyanidins upon heating in acidic media. The latter class of flavonoids is responsible for the pigments that give the dark red, blue, and purple colors [33]. The most common members of the PAs are the procyanidins (PCs), composed of the monomeric flavan-3-ols (+)-catechin and/or (-)-epicatechin (Figure 1). These elementary units are usually linked by C-C and occasionally by C-O-C bonds.

The phenolic polymers, formed by enzymatic and/or chemical transformation of simple flavanols, PAs and other phenolic compounds (during plant tissue damage, post-harvest storage and processing), are termed tannin-like compounds and comprise the third group of tannins [31]. These new compounds share common structural features with tannins: high molecular mass and similar number of phenolic rings per mass unit [31]. They are formed by irreversible oxidative reactions catalysed by enzymes such as polyphenoloxidases or metal ions. They also share common properties, the most evident being astringency. These are most likely to be the constituents of pre-fabricated animal feed.

The nature of tannin-protein interaction in biological and food domains has been the subject of several studies [34,35,36,37]. The nature of the interaction probably depends on the nature of the polyphenol, its size, its stereochemistry, the nature of the protein, and the medium in which the interaction (ionic strength and pH) takes place [38,39,40]. They have been extensively studied using a variety of techniques and experimental models in relation to astringency and antinutritional effects and are reported to generate insoluble and soluble complexes [34,36,39,40,41]. Potentially, such interaction could occur via covalent or ionic bonds, hydrophobic interaction, or hydrogen bonding. Polyphenols are prone to oxidation and give rise to *ortho*-quinones which are highly reactive intermediates that potentially could than result in tannin-protein covalent crosslink [34,35]. Thus far, different mechanisms involving hydrogen bonds and hydrophobic interactions have been made responsible for precipitation of condensed and hydrolyzable tannins [34]. Hydrophobic effects have been considered in various papers as the main driving forces toward association, probably enhanced by hydrogen bonding between the phenolic groups and the protein [34,38,41,42]. The stability of protein–tannin complexes has been stipulated to increase with the number of bound tannins and presumably with the number of repeated amino acid sequences [36]. In general it has been found that tannin binding proteins are hydrophobic, have large molecular mass, high proline content and lack secondary structure [39,40,42]. 

Due to these mentioned characteristics of tannins and to their feature of a high affinity for proteins, saliva presents a particular importance in all the process of tannin oral perception and ingestion. The binding of these polyphenols with salivary proteins and/or oral mucosal proteins is related to their characteristic astringency [43,44]. Besides the most studied salivary PRPs [34], recent studies suggest an involvement of other salivary proteins in tannin oral perception [45,46,47]. This issue will be further developed in Section 4.

### 3.2. Tannin effects on ruminant nutrition

Tannin effects on ruminant nutrition have been studied for several years, and are often seen only in terms of their negative impacts on intake and production: decreased nutrient utilization, particularly protein [48]; decreased palatability and consequently the amount of food ingested; decreased digestibility [6,7,49]; volatile fatty acids production reduction, and decreased digestibility of organic matter and fiber [50]; damage of kidney and liver [8]; tissue damage in the rumen, intestine ulceration and morphological changes at the *microvilli* level [51]. However, besides these anti-nutritional and toxic effects, there is an increasingly awareness of tannin’s beneficial roles on animal nutrition and health [52], namely influences on the cell signaling pathways [53], anti-oxidative effects [54], and anti-helmintic [55,56] and anti-microbial [57] activities. In ruminants a particularly important positive effect of tannins is dietary protein protection from ruminal microflora attack [58,59]. Due to the binding of tannins to dietary protein, and also to a reduction in the activity of a large proportion of microflora, there is an increased rate of amino acid absorption in the intestine, which improves the utilization of nitrogen by ruminants [60]. As well as binding to protein, tannins can also bind to carbohydrates, leading also to a reduction in ruminal gas production [6,60]. Due to a combination of these activities tannins can be associated with improvements in animal growth and productivity and consequentially minimization of effects to the environment. 

The presence of high amount of PSMs in certain plants may limit their intake below the animal requirements for energy and protein. Consequently herbivores tend to defend themselves by eating a variety of plant species that contain different types and levels of compounds, rather than one sole species. This is due to the fact that each of the different types of PSMs affect the organism in different ways and are detoxified by different complex mechanisms. Such a behavior may imply a key mechanism for reducing the toxicity associated with a particular type of metabolite [61]. Concerning the presence of tannins in pastures, if animals have diverse plant species available they are able to tolerate it better. It was observed that sheep eat more when offered three foods that contain predominantly terpenes, tannins and oxalates than when offered food with only one or two of such PSMs [62]. Comparison of intake of shrubs containing only tannins or saponins with the intake of shrubs containing both phytochemicals indicates that goats consume more biomass when fed with shrubs with both classes of compounds [63]. This complementary relationship was also referred on cattle fed on tannin, saponins and alkaloids containing fodder [64]. 

### 3.3. Tannins and sensory perception

Animal feed selection depends heavily on the palatability of the feed. Tannins are usually associated to a decrease in palatability, and consequently discourage grazing [65]. High tannin levels reduce preference of plants by cattle, sheep and goats [49]. Several studies suggest that contents of condensed tannins higher than 50g/kg dry matter (DM) significantly reduce voluntary feed intake, in most ruminants, while medium or low levels seems to have a minor effect (reviewed in [66]).

A tannin sensory cue often associated with decreases in palatability is astringency. This mechanical sensation is generally assumed to result from phenol/salivary protein interactions [43,44]. Astringency is a puckering or drying sensation, not localized in any one part of the mouth, and that typically develops and dissipates slowly [67]. One accepted theory suggests that tannin-salivary protein interaction results in the formation of insoluble precipitates which stimulate mechanoreceptors in the mouth. Based on this, it has been assumed that astringency perception is positively related to the salivary concentration of proteins with tannin-binding capacity [39,68]. In addition, several authors have suggested that astringency is a result of the loss of lubrication properties of the salivary film that protects oral mucosa. The binding of tannins to mucins and other glycosylated proteins would lead to modifications of viscous elastic properties of this film [69,70,71,72,73]. As such, differences in salivary constituents may account for differences in astringency perception.

A bitter taste was also proposed for tanniniferous diets [74]. Additionally, the development of physical discomfort, by consuming tannins, evokes an aversion towards foods containing these compounds, which was suggested to be mediated by emetic mechanisms in the nervous system [9,27]. In other words, post-ingestive experience is paired to sensorial characteristics and consequently decreases the acceptance [9,27]. Although the exact mechanism is poorly understood and results are sometimes conflicting, the effect was observed to differ according to animal species. Sheep and cattle seem to be more sensitive to tannins than goats. The perceived sensitivity to tannins could be related to species different ability to tolerate and orally detect these compounds.

Although oral sensitivity in ruminant species is not well documented, results from experiments made thirty years ago, on taste sensitivity in cattle, sheep and goats, have demonstrated that there are differences between species regarding gustatory chemoreception [75]. Goatcher and Church [76] observed that when a bitter solution, such as quinine, is presented, goats can detect the bitter taste at lower concentrations than cattle or sheep. However, this was not immediately translated in rejection behavior and at low concentrations it seems even that they showed a preference for the bitter taste. With increased bitter concentrations the preference declined and for high concentrations these animals started to show rejection. Despite the higher taste thresholds for bitter taste, sheep showed a stronger rejection of bitter compounds compared to goats [76]. A suggested explanation is that goats may need high bitter taste sensitivity in order to choose nutritive meals from a mixture of plants, when in favourable conditions. At the same time, in hard conditions, where browse is abundant, they need not to reject every bitter compound, at the risk of being underfed [77]. Besides this information on basic tastes, also considering the global oral food perception, there are differences among ruminants. Robertson *et al*. [78] found that sheep and goats presented similar patterns of response to different food flavours, but differed in the level of response. Previous results from our group (data not published) also presented evidences that sheep and goats perceive tannins differently. Moreover, we observed that for both species, the behavioral response presented to pellets flavoured with hydrolysable tannins was not the same as the one presented to pellets flavoured with condensed ones. It was not possible to conclude if such a differential response was due to different taste sensitivity and/or a different astringency perception. Taken these results together, it is possible that one reason for differences in the levels of tannins acceptance by the different species may be their ability and/or tolerance to tannins oral sensations. However, this is an area that still needs further investigation. 

## 4. The Role of Saliva and Salivary Proteins in Tannin Ingestion

The basic role of saliva is the protection and maintenance of the integrity of the upper part of the digestive tract, through the following important functions: lubrication; buffering action and clearance; maintenance of tooth and mucosal integrity; antibacterial and antiviral activity as well as taste and digestion [79]. Three pairs of major exocrine glands (parotid, submandibular, and sublingual), plus numerous minor salivary glands, are the responsible for salivary protein secretion. Parotid glands are constituted by serous acinar cells, which produce a thin watery saliva, containing a diversity of protein types, among which proteins showing high phenol-binding capacity (PRPs, amylases, histatins, cystatins) [34,80]. Submandibular glands consist of both serous and mucous acinar cells, being the mucous cells responsible for a viscous mucin-rich secretion mainly responsible for mouth lubrication [81]. Sublingual glands are almost only composed by mucous cells.

Saliva is a fluid with a major importance for diet adjustment. In fact, it serves as a physiological buffer against variations between the external and internal milieus. Such variations may be reflected in different salivary protein profiles resultant from different dietary habits [46]. It has been proposed that saliva protein composition varies considerably among species, reflecting diverse diets and modes of digestion. Salivary glands are under nervous control and the composition of their secretions is rapidly changed over a wide range of different stimuli [82]. There are species particularities concerning salivary glands specific distribution, size and weight, which have been related to differences in dietary niches among ruminants [83,84].

Saliva has been referred as a first line defense against tannin ingestion. The presence of proteins with a particular ability to bind tannins [tannin-binding salivary proteins (TBSPs)] has been reported in the saliva of several animal species (for a review see [37]). A large number of studies link the occurrence of TBSPs to the levels of tannins present in the individual’s normal diet, arguing that species with low tannin content in their natural forage have little or none of such salivary proteins [85,86]. For browsers it was proposed the constant presence of TBSPs, whereas in grazers they would be inexistent, and in species such as deer (*Odocoileus hemionus*) for which the content of tannins in diet changes seasonally, their production would occur when consuming plants rich in tannins [5,85].

The most studied TBSPs are the proline-rich proteins (PRPs). These are salivary proteins found constitutively in human saliva [34], and which are induced in animal species such as rats [87] and mice [88] following tannin consumption. Their presence was also reported in primates [89] and pigs [90]. There are three types of salivary PRPs: the acidic, the glycosylated, and the basic proteins. Acidic PRPs have the main function of controlling calcium levels, whereas glycosylated PRPs aid lubrication of food boluses [34,67,91]. Until now, the biological function of basic PRPs was not completely understood, being proposed that these proteins have an important role in binding to polyphenols [30,34]. Basic proline-rich proteins have a similar sequence across a wide range of species, being dominated by the amino acids proline, glutamine, and glycine, which repeats to give a protein which is typically extended in solution [67]. PRPs have a particular high capacity to bind tannins, and the complexes formed appear to be stable across the whole pH range of digestive tract, allowing tannins to pass intact through it and to be excreted [34]. It was suggested that the presence of TBSPs might also cancel the negative effects of tannins on palatability, and consequently on feed intake, improving the utilization of plants containing such compounds [5,77,85,92].

Besides PRPs, in humans, the salivary histatins were observed to have affinity to tannins [42]. However, in other species the presence of these proteins and their role in tannin ingestion has not been deeply investigated. Salivary amylase also presents high affinity for tannins [93]. Studies from our group presented evidences of changes in expression levels of amylase isoforms in response to increased levels of dietary tannins [45,94]. One hypothesis suggested was that, despite the primary biological function of salivary α-amylase being the digestion of polysaccharides, this increase may reflect an adrenergic stimulation action by tannins, rather than a specific salivary response as a defense mechanism. Another possible explanation is that being tannins potent inhibitors of salivary amylase [94,95], the increase in this protein secretion may aim to counteract the decrease in its activity in the mouth, similar to the pancreatic response that occurs following decreased amylase activity in the gut induced by tannin ingestion [96]. However, until now, the presence of amylase was not observed in ruminant species and as such the affinity of this salivary protein to tannins will not be detailed in the present review.

Concerning the three ruminant species focus of this review, studies are not conclusive regarding the role of saliva as a defense mechanism against dietary tannins ingestion (Table 1) Some suggest that sheep, cattle and goat saliva does not have a great ability to bind tannins [97]. Others reported that the saliva of browsers contains higher protein concentration and has a greater tannin-binding capacity than saliva from the grazer species cattle and sheep [5,85]. Accordingly, because cattle and sheep predominantly consume grass diets virtually free of tannins, they would not need to produce TBSPs [85]. However, Robbins *et al.* [5] suggested the possibility of these salivary proteins in sheep being induced by consumption of plants rich in tannins. In the case of cattle, it has not been observed an increase in the production of PRPs in response to tannin ingestion [98]. However, Burritt *et al.* [99] reported a marked reduction of tannins in samples collected from esophageal fistulae of cattle consuming tannin-rich browse, suggesting a salivary defense mechanism in this specie. Also, salivary proteins other than PRPs, but with high affinity for polyphenols, have been suggested to be present in cattle saliva [98].

**Table 1:** List of studies in which the presence of Tannin-binding salivary proteins (TBSPs) was referred for sheep, cattle and goats.

Specie	Presence of TBSPs		Reference
	Constitutive^1^	Induced by tannins^2^	
Sheep (*Ovis aries*)	No	No	[5,85,117,118]
	Yes		[107]
	(unidentified^3^)		
		Yes	[118]
		(unidentified)	
Cattle (*Bos taurus*)	No	No	[5,85,97]
		Yes	[98]
		(other type^4^)	
Goat (*Capra hircus*)	No		[97,103,111,118]
	Yes		[99,102,107]
	(unidentified)		
		Yes	[118]
		(unidentified)	

^1^ Presence/absence in saliva produced under consumption of each species regular diets; ^2^ Presence/absence in saliva produced following stimulation with a tannin-rich diet; ^3^ The presence of TBSPs was reported but the detailed characteristics have not been investigated; ^4^ The presence of TBSPs, which are not PRPs, has been reported.

In what refers to goats, the controversy in the literature, about the presence of tannin-binding proteins in saliva, is even higher than for the grazer species. According to the “niche theory” (i.e., the relational position of a species in its ecosystem), goats should produce TBSPs, since they base their diet in tannin-rich browse [22,100]. Some authors suggested that goat produce more protein-rich saliva during eating than sheep [101], and have salivary defense mechanisms for tannin-rich browse [99,102]. On the other hand, other authors [e.g. 103] did not detect tannin-binding proteins in the saliva of goats fed a tannin-rich diet.

Parotid has been the salivary gland more frequently associated to the synthesis and production of TBSPs. In rodents, tannin ingestion can induce parotid hypertrophy [104,105]. In roe deer, the affinity of mixed saliva for both tannic acid and quebracho condensed tannins was observed to be only 50–65% of that of parotid saliva [106]. Gilboa (cited by [7]) found that the parotid saliva of goats was relatively rich in proline (6.5%), glutamine (16.5%) and glycine (6.1%). This author also observed that the concentration of parotid saliva of goat fed a tannin-rich diet was higher than from goats maintained on diets low in these PSMs. The presence of TBSPs in goats and sheep parotid glands was also reported [107]. Recently, using proteomic techniques, we were unable to identify PRPs in sheep or goat parotid saliva, neither constitutively [46,47,108] nor when feeding on a tannin-enriched diet [47]. Nevertheless, we observed some changes in parotid salivary proteome induced by tannin ingestion [47], and as such the involvement of saliva in the consumption of tannins, by these species, may not be discarded. Consumption of quebracho tannins resulted in the increase in the expression of both the protein cytoplasmic actin 1 and the protein annexin A1, in both species. These proteins may not act as TBSPs, but may be the consequence of an increased salivary gland function, induced by tannins. For example the increase of actin, which is a protein from the cytoskeleton, may be related to the particular “apocrine-like” mode of salivary secretion presented by ruminants [109] and to the fact that an increased salivation rate might result in increased cytoplasmic content in saliva. Additionally, annexin A1 is a protein already observed to be increased in humans after tasting bitter/sour solutions [110]. To our knowledge, an association of this protein to tannin consumption has not been reported.

A recent study [111] failed in to find proteins that bind tannic acid or quebracho tannins in goat saliva. Moreover, these authors observed that goat mixed saliva present a higher affinity to tannins than parotid saliva. One possible explanation for the discrepancy of results concerning the involvement of each salivary gland in tannin consumption may be the differences in the molecular types of tannins tested across the different studies [111]. Nevertheless, we observed some changes in rodents’ submandibular glands induced by a tannin-enriched diet [105]. A possible (although less explored) explanation, for a higher affinity of goats mixed saliva to tannins, is a potential role of salivary mucins. In fact, a 2-step salivary protein/dietary phenol interaction has been hypothesized in which saliva is considered to be composed of 2 different phases: a thin dynamic film coating the internal oral surfaces and an adsorbed layer of proteins on the hard and soft tissues [112]. The first step of protein/phenol interaction might involve the dynamic film consisting of proteins with the highest phenol-binding affinity (PRPs, amylases, cystatins, and histatins), as we referred before. The second step might be based on phenol interactions with the adsorbed glycoprotein layer (among which salivary mucins) with a consequent oral cavity delubrication and astringency elicitation. Van Soest [113] referred that goats may secrete higher levels of salivary mucins than sheep or cattle, and for that reason they can be more tolerant to high levels of dietary tannins. Moreover, [114] reported that in comparison to bovines, camel saliva contains a varying amount of high molecular weight mucin glycoprotein that confers protection to the mucosa of the digestive tract from plant tannins. Assuming that, such a higher tannin affinity in mixed comparatively to parotid saliva might be related to mucins and non-parotid derived glycoproteins. However, this hypothesis need to be further explored, since the role of salivary mucins in astringency perception is not completely clear. Whereas mucins have been shown to bind polyphenols and proposed to being involved in astringency [115], a recent study, in human saliva, did not found a correlation between salivary mucins concentration and perceived astringency [116]. 

## 5. Conclusions Remarks and Future Prospects

A chief characteristic of animal production in the Mediterranean region is the huge variation in plant communities, biomass production and chemical composition. This stands for natural pastures found under tree canopy of oak woodlands, coexisting with patches of shrublands, and with pastures resulting from agricultural land use such as diverse as olive trees orchards and vineyards. The variation in plant communities is increased by extreme quantitative and qualitative seasonal changes. The resulting diversity in available plants and plant parts nutrient levels and PSMs are powerful determinants of diet selection. To face it, different animal species use different trophic strategies, changing voluntary intake, food items ingested, or modifying digestive physiology. Grazing herbivores meet their nutritional goals by prioritizing certain nutritional parameters when choosing the types and quantities of different foods, namely by avoiding or regulating intake of plant secondary metabolites. Tannins are polyphenols, which directly or indirectly affect intake and digestion. They are the primary source of astringency in plants, which results from binding to proteins, forming soluble or insoluble complexes. The nature of the interaction is greatly dependent on the structure of the polyphenols and the proteins involved. However, the exact constituents and mechanisms responsible for tannin oral sensations are not completely known. Salivary PRPs have been the most studied family of TBSPs, but recent evidence points to the involvement of other salivary proteins on ingestive behavior. Due to the relevance that polyphenols consumption has on nutrition and health, this is a research area that needs further investigation. Future prospects include to identify the salivary proteins that may complex tannins and to deeply understand the type of the interactions involved and to gather more information about structure-function relationships. Altogether these data will contribute to a better use of Mediterranean ecosystems and natural resources.
